# Post-Translational Modifications of PCNA in Control of DNA Synthesis and DNA Damage Tolerance-the Implications in Carcinogenesis

**DOI:** 10.7150/ijbs.64628

**Published:** 2021-09-23

**Authors:** Siyi Zhang, Tingting Zhou, Zhuo Wang, Fei Yi, Chunlu Li, Wendong Guo, Hongde Xu, Hongyan Cui, Xiang Dong, Jingwei Liu, Xiaoyu Song, Liu Cao

**Affiliations:** 1 Institute of Health Sciences, China Medical University, Shenyang, Liaoning Province, 110122, PR China.; 2 College of Basic Medical Science, Key Laboratory of Cell Biology of Ministry of Public Health, Key Laboratory of Medical Cell Biology of Ministry of Education, Liaoning Province Collaborative Innovation Center of Aging Related Disease Diagnosis and Treatment and Prevention, China Medical University, Shenyang, Liaoning Province, 110122, PR China.

**Keywords:** replication, DDT, ubiquitination, phosphorylation, acetylation, SUMOylation, NEDDylation, ISGylation

## Abstract

The faithful DNA replication is a critical event for cell survival and inheritance. However, exogenous or endogenous sources of damage challenge the accurate synthesis of DNA, which causes DNA lesions. The DNA lesions are obstacles for replication fork progression. However, the prolonged replication fork stalling leads to replication fork collapse, which may cause DNA double-strand breaks (DSB). In order to maintain genomic stability, eukaryotic cells evolve translesion synthesis (TLS) and template switching (TS) to resolve the replication stalling. Proliferating cell nuclear antigen (PCNA) trimer acts as a slide clamp and encircles DNA to orchestrate DNA synthesis and DNA damage tolerance (DDT). The post-translational modifications (PTMs) of PCNA regulate these functions to ensure the appropriate initiation and termination of replication and DDT. The aberrant regulation of PCNA PTMs will result in DSB, which causes mutagenesis and poor response to chemotherapy. Here, we review the roles of the PCNA PTMs in DNA duplication and DDT. We propose that clarifying the regulation of PCNA PTMs may provide insights into understanding the development of cancers.

## Introduction

Accurate DNA replication is essential for appropriate cell division. Human cells suffer from various “stress” at the replication fork, where the stress derives from replication obstacles and sources of damage. The replication obstacles contain re-replication, misregulated origin licensing, nucleotide depletion, and oncogene activation. The damage sources include reactive oxygen species (ROS), ultraviolet (UV), ionizing radiation (IR), and DNA damaging chemotherapy drugs. During replication, DNA synthesis stalls when replication machinery encounters lesions. Persistent replication stalling will cause DNA double-strand break (DSB). Because the failure of post-replication DNA damage repair leads to cell death or genomic instability, organisms evolve DNA damage tolerance (DDT) mechanisms to bypass DNA lesions. DDT pathways mainly consist of the error-prone translesion synthesis (TLS) and error-free template switching (TS) pathway.

Proliferating cell nuclear antigen (PCNA) plays a central role in DNA synthesis and DDT. PCNA serves as a moving platform recruiting various factors for replication or DDT. A conserved motif named PCNA-interacting protein box (PIP box) has been found in PCNA-binding partners. The core element is a consensus sequence Q1-x2-x2-h4-x5-x6-a7-a8, where “h” represents hydrophobic residues (L, M, I, or V) and “a” being aromatic amino acids (F, Y, or H) 1.

**Figure 1:**
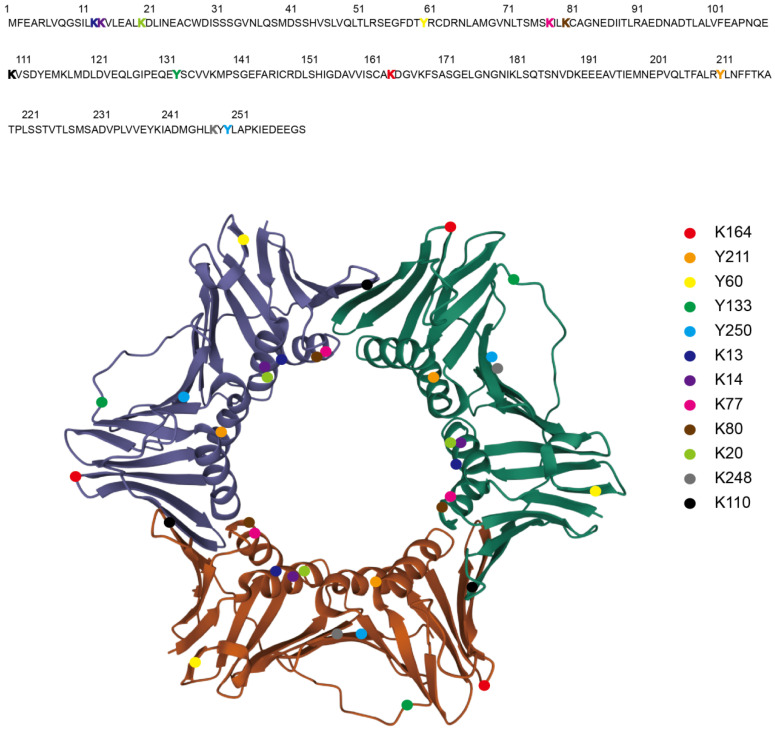
** The Structure of PCNA Homo-trimer and the PTMs Sites.** The crystal structure of *Homo sapiens* PCNA homo-trimer is shown as each monomer with different color (Protein Data Bank: 1VYM) 158. The PTMs sites are highlighted in the amino acid sequence as colored characters (upper) and in the homo-trimer as colored balls (lower). Ubiquitination (K164), SUMOylation (K164), phosphorylation (Y211, Y60, Y133, Y250), acetylation (K13, K14, K77, K80, K20), methylation (K248, K110).

**Figure 2:**
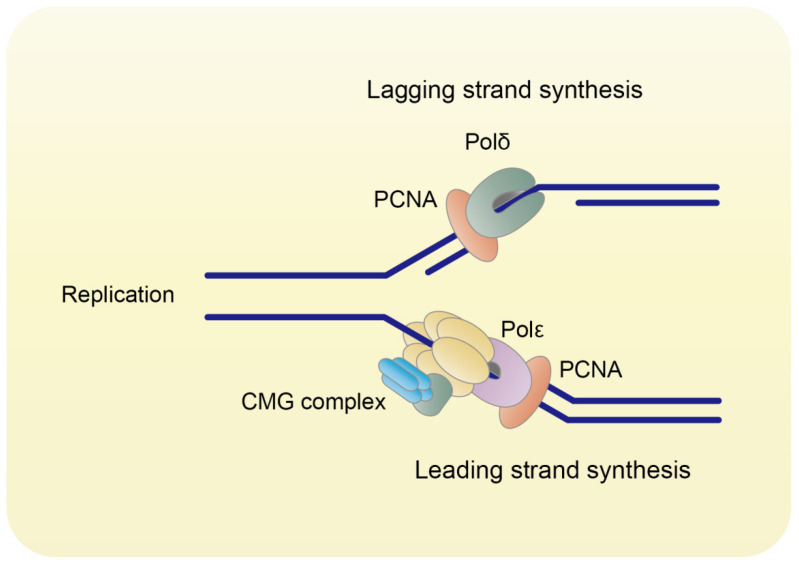
** PCNA orchestrates DNA replication.** PCNA binds to Polδ and Polε to orchestrate DNA lagging strand and leading strand synthesis, respectively.

During DNA replication, three monomers of PCNA form a ring-shaped homo-trimer (Figure 1), which encircles DNA and recruits B-family DNA polymerases (Polδ and Polε) to carry out the high fidelity DNA synthesis (Figure 2) 2. In eukaryotes, Polδ is dominant over Polε in association with PCNA 3. Polδ is responsible for the lagging strand synthesis, while Polε acts on the leading strand for replication 4, 5.

**Figure 3:**
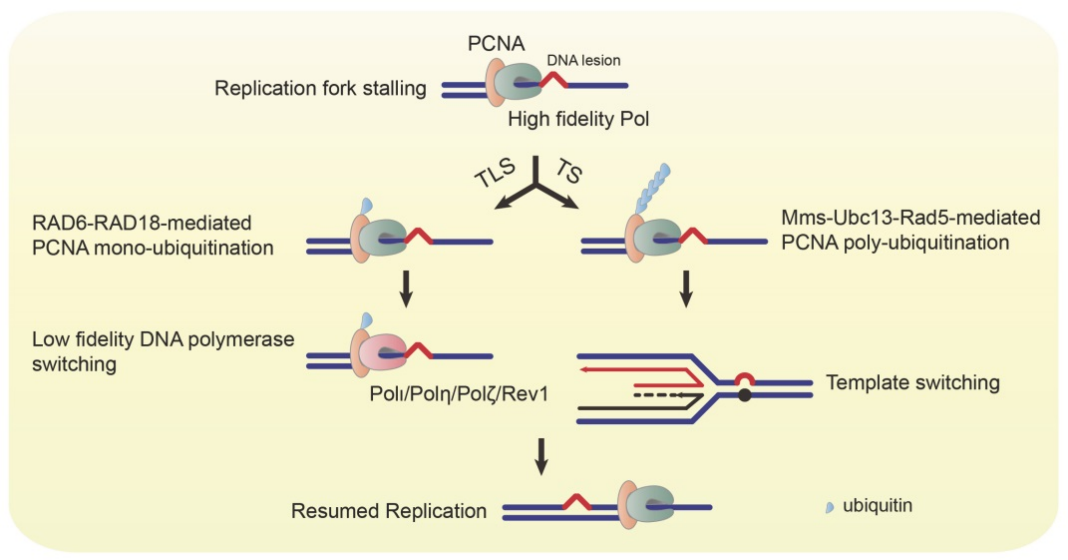
** Ubiquitinated PCNA mediates TLS and TS.** When the replication fork encounters a DNA lesion, the replication machinery stalls, and PCNA is mono-ubiquitinated by the RAD6-RAD18 complex. The mono-ubiquitinated PCNA switches recruitment of low fidelity DNA polymerases (Polι, Polη, Polζ, and Rev1) to bypass the lesion. The mono-ubiquitinated PCNA can also be poly-ubiquitinated by the Mms-Ubc13-Rad5 complex. The poly-ubiquitinated PCNA mediates the template switching to resume replication.

The replication fork stalls when the replication machinery confronts DNA lesions, and PCNA is mono-ubiquitinated at lysine residue (K) 164 by RAD6-RAD18 complex 6. The mono-ubiquitinated PCNA switches its association from high fidelity polymerases to low fidelity ones, such as Polι, Polη, REV1, and Polζ 7-9. These polymerases help the replication fork bypass the damaged bases to continue DNA synthesis (Figure 3) 10-14. However, every coin has two sides. The TLS pathway ensures uninterrupted replication and protects cells from DSB, while it also increases the frequency of mutations due to the low accuracy of TLS polymerases. In addition to the RAD6-RAD18 complex, the K63 poly-ubiquitination of PCNA at K164 requires the Mms2-Ubc13-Rad5 complex. The poly-ubiquitinated PCNA triggers the error-free TS (Figure 3) 15. Thus, dysregulation of replication and the TLS/TS pathway will result in genomic instability and tumorigenesis.

Post-translational modifications (PTMs) possess various functions such as modulating protein activity, localization, stability, and interactions 16. This review discusses the recent studies about the PTMs in regulating PCNA functions and the implications in tumor development.

## Ubiquitination

Ubiquitin is an 8.5 kDa small protein with 76 amino acids. Protein ubiquitination, discovered in the early 1980s, is sequentially catalyzed by the ubiquitin system containing an ubiquitin-activating enzyme (E1), an ubiquitin-conjugating enzyme (E2), and an ubiquitin ligase (E3). Initially, studies reported that ubiquitination mainly guides the tagged protein for 26S proteasome degradation.

As the investigation develops in-depth, proteins are either modified with a single ubiquitin (mono-ubiquitination) or polymeric ubiquitin chains (poly-ubiquitination). Mono-ubiquitination serves as a signal for DNA damage response, such as mono-ubiquitinated H2AX/H2A/H2B and PCNA 17. Additionally, it acts as a critical step for subsequent assembly of poly-ubiquitination 18. Based on the different lysine (K) sites of the ubiquitin conjugation, poly-ubiquitination is diverse in various types, including K6, K11, K27, K29, K33, K48, and K63 poly-ubiquitination 19, 20. Different ubiquitin linkages result in a diversity of cellular signals. The most well-studied types of poly-ubiquitination are the K48- and K63-ubiquitin chains. The K48 poly-ubiquitination labels substrates for proteasome degradation 21, while the K63 poly-ubiquitination participates in DNA damage response 22. Protein ubiquitination is a reversible process that can be influenced by the ubiquitin-cleavage activity of deubiquitinating enzymes (DUBs).

In response to stalled replication forks, PCNA is mono-ubiquitinated by a sequential process mediated by an E1, RAD6 (E2), and RAD18 (E3) 6. The human homolog of RAD6 is HHR6A and HHR6B 23. The human RAD18, an approximately 54kDa protein consisting of 484 amino acids, is corresponded to yeast RAD18 24. PCNA is mono-ubiquitinated specifically at K164 during TLS 6, 12. When PCNA is mono-ubiquitinated, it switches its interaction from high fidelity Polδ or Polε to low fidelity TLS polymerases (Polη, Polι, Polκ, Polλ, Polζ, or REV1). Then, these TLS polymerases proceed to bypass the lesions 10-14, 25.

High expressions of RAD6 and RAD18 are positively related to the development and therapy resistance of various tumors 26-36. Sanders *et al*. identified the small molecular inhibitors of RAD6. Among them, SMI#9 shows a potent inhibition effect on the proliferation and migration of breast cancer cells 37. It has been demonstrated that SMI#9 attenuates chemotherapy agent-induced PCNA mono-ubiquitination and enhances chemosensitivity of triple-negative breast cancer (TNBC) or ovarian cancer 35, 36, 38. Inhibition of RAD18 has also been found to suppress gastric cancer progression and sensitize gastric cancer to chemotherapy via reduction of PCNA mono-ubiquitination 31. However, the small molecular inhibitors of RAD18 have not been designed, which needs further studies to find effective ones for tumor treatment.

The K63-linked poly-ubiquitination of PCNA at its K164 site is involved in error-free lesion bypass via TS 15. In addition to the RAD6-RAD18 complex, PCNA poly-ubiquitination requires RING-finger E3 ligase Rad5, which recruits Ubc13 and Mms2 as an E2 complex 39. The human homolog of Rad5 contains the helicase-like transcription factor (HLTF) and the SNF2 histone linker PHD RING helicase (SHPRH) to mediate PCNA poly-ubiquitination 40. The mechanism of poly-ubiquitinated PCNA regulating lesion bypass has not been fully understood. One possibility is that poly-ubiquitinated PCNA may induce replication fork reversion to promote a recombination-like process using the undamaged DNA duplex as a template.

DNA binding is critical for PCNA mono-ubiquitination, as mono-ubiquitination of free PCNA is inhibited by the overabundance of free replication protein A (RPA) through its interaction with RAD18 under native conditions 41. RPA, the main ssDNA-binding protein, is required for DNA replication, recombination, DNA damage repair, cell cycle checkpoints, and DNA damage checkpoints 42. RPA undergoes a conformational change during replication stress when it binds to ssDNA and forms filaments at TLS sites. RAD6-RAD18 complex is recruited to the RPA filaments and then promotes the mono-ubiquitination of PCNA on DNA 41, 43.

The chromatin microenvironment modulated by various factors may be crucial for the regulation of PCNA ubiquitination. The chromatin structure modulated by histone modifications and nucleosome remodeling is essential for the control of replication, transcription, and DNA damage repair 44-46. The cells with the mutations of histone H3/H4, which participated in packaging DNA into chromatin, showed a reduced level of PCNA mono- and di-ubiquitination after treating with methyl methanesulfonate (MMS) or UV. Accordingly, the H3/H4 mutants exhibited decreased MMS-induced mutagenesis compared to wild-type ones 47.

WRN protein is one member of the RecQ helicase family. The mutation of the *WRN* gene causes Werner Syndrome (WS) with premature aging and predisposition to cancer 48, 49. In response to DNA damage agents, WRN forms discrete nuclear foci only during the S phase 50, 51, implying the essential roles of WRN in replication-post DNA damage repair. WRN interacts with PCNA in the absence of DNA damage. ATM-mediated phosphorylation of WRN causes its degradation in response to DNA damage. This degradation results in the release of PCNA from WRN that probably promotes PCNA ubiquitination. The WS and WRN knockdown cells show high-level ubiquitination of PCNA even without DNA damage 51. These results indicate that WRN may inhibit aging and carcinogenesis via PCNA ubiquitination regulation. A small molecular inhibitor NSC19630 against WRN effectively inhibits tumor cell growth and induces apoptosis via impairing the response to DNA damage and replication stress 52. Novel small molecular inhibitors targeting WRN have also been screened out, prohibiting the proliferation of cancer cells 53. However, the underlying mechanisms require further investigation.

The human PTIP protein, the homolog of the *Xenopus* Swift with six BRCT domains participating in DNA damage repair, is an adaptor for ATM/ATR kinase 54-56. The investigation from PTIP/Swift deleted mouse and yeast models reveals that PTIP/Swift plays a significant role in DNA damage repair during replication 57, 58. Depleting PTIP/Swift in *Xenopus* and human cells compromised the mono- and di-ubiquitination of PCNA in response to UV 59, raising the potential role of PTIP/Swift in DDT.

RNF8 is an E3 ligase and a key DNA damage response factor 60. It was identified as a novel ligase for mono- and poly-ubiquitination of PCNA using UbcH5c and Ubc13/Uev1a as an E2 ubiquitin-conjugating enzyme, respectively 60. Similar to the RAD6-RAD18 complex, RNF8-UbcH5c-mediated mono-ubiquitination of PCNA serves as a substrate for poly-ubiquitination 60. In medulloblastoma cells, knockdown of RNF8 inhibited IR-induced PCNA ubiquitination while enhanced cell cycle arrest and apoptosis 61. However, the deletion of RNF8 barely affected PCNA ubiquitination in DT40 chicken cells after exposure to UV 62. The effects of RNF8 on PCNA ubiquitination call for further research in different species of cells.

The insulin-like growth factor (IGF) signal pathway primarily functions in cell proliferation, survival, apoptosis, differentiation, metabolism, and migration 63. It also participates in PCNA ubiquitination. After exposure to UVB, the skin of individuals over the age of 65 shows an increased level of PCNA mono-ubiquitination 64. The geriatric skin contains a decreased expression of IGF-1 and reduced activation of IGF-1 receptor (IGF-1R) 65. Both deprivation of IGF-1 and inhibition of IGF-1R result in elevated UVB-induced PCNA mono-ubiquitination in human skin and keratinocytes 64. Exposure to UV is the primary cause of non-melanoma skin cancer, which possesses mutagenic photoproducts in DNA. These findings suggest that the IGF signaling pathway may prevent UV-induced carcinogenesis by regulating PCNA ubiquitination.

Phosphorylation kinases are emerging as targets attracting the most attention for cancer therapy development 66, 67. By screening the library of kinase inhibitors, the inhibitor targeting pro-survival kinase Akt has been shown to impair the mono-ubiquitination of PCNA in cells after UV exposure. Persistent inhibition of Akt resulted in the blockage of TLS Polη recruitment to DNA damage sites and further perturbation of DNA replication processivity, which led to synthetic lethality in homologous recombination (HR)-deficient cells 68. However, this study did not unravel whether Akt could phosphorylate PCNA to regulate its ubiquitination. Nevertheless, it points to the field of modification crosstalk targeting PCNA for TLS regulation.

The deubiquitination enzyme USP1 reversely regulates PCNA ubiquitination in the S phase, which is critical to prevent unwanted mutagenesis. After the cells are exposed to UV, USP1 is downregulated by autocleavage and accompanied by upregulation of PCNA ubiquitination 69. USP1-induced deubiquitination of PCNA is positively regulated by ELG1, the component of the RFC complex. ELG1 directs USP1-UAF1 complex for PCNA 70. USP1 is upregulated in BRCA1 deficient tumors. The USP1-mediated restriction of PCNA ubiquitination protects replication fork in BRCA1-deficient cells because persistent ubiquitination of PCNA recruits Polκ and REV1, which contributes to replication fork instability. Thus, the USP1 inhibitor may be beneficial for a subset of PARP inhibitor-resistant BRCA1-deficient tumors 71.

Kashiwaba *et al*. have identified another DUB USP7, which is responsible for the reduction of H_2_O_2_-induced PCNA mono-ubiquitination. Suppression of USP7 increases H_2_O_2_-induced mutagenesis in cells 72. It has also been found that USP7 removes K63 poly-ubiquitination of PCNA 73. Surprisingly, USP7 can also facilitate UV-induced mono-ubiquitination of PCNA via deubiquitinating and stabilizing Polη 74, 75. Hence, further studies need to clarify whether USP7 affects PCNA ubiquitination in cells under different stresses. Moreover, ubiquitinated PCNA can also be reverted by Ubp2, Ubp10, Ubp12, and Ubp15 in the S phase 76-78.

Thus, timely regulation of ubiquitination and deubiquitination of PCNA is essential to limit DDT and ensure appropriate DNA replication.

## SUMOylation

The SUMOylation is a reversible and dynamic PTM that conjugates small ubiquitin-related modifier (SUMO) to the lysine of target proteins. The nascent SUMO is proteolytically cleaved by SUMO-specific proteases (SUPs) to expose the C-terminal glycine-glycine (GG) motif. The SUMO E1 enzyme activates the mature SUMO. Then, it is transferred by the SUMO E2 and E3 enzyme to the substrate. SUMOylation is involved in several biological processes, including protein-protein interactions, protein-DNA binding, subcellular localization, transcriptional regulation, and DNA repair 79. The aberrant regulation of SUMOylation is frequently related to cancer, cardiac disease, and neurodegenerative disease 80-82.

The human SUMO molecules contain four paralogs (SUMO1-4). PCNA can be conjugated with both SUMO1 and SUMO2 83-85. PCNA is SUMOylated at K164 and K127. Distinctively from K164 being both ubiquitinated and SUMOylated, K127 is only SUMOylated but cannot be ubiquitinated 6. By co-expressing ectopic PCNA with SUMO1, SUMO2, and SUMO3 in human cells, it was found that SUMO1 is predominantly conjugated to PCNA, while SUMO2 or SUMO3 is weakly attached to PCNA even with UV treatment. Furthermore, the *in vitro* PCNA SUMOylation assay revealed that the E1 SUMO-activating enzyme (SAE1/SAE2), the E2 SUMO-conjugating enzyme (Ubc9), and the E3 SUMO-ligases (Pias1, Pias2, Pias3, and Pias4) participate in SUMO1 conjugating to PCNA 84.

PCNA SUMOylation occurs during the S phase or in the presence of DNA damage. In yeast, the SUMOylated PCNA cooperates with the helicase Srs2 to inhibit HR repair during the S phase 86. The recruitment of Srs2 by SUMOylated PCNA at replication sites disrupts Rad51 nucleoprotein filaments and dissociates Polδ/Polη from the repair synthesis machinery 87. This mechanism controls the spontaneous HR 88, which contributes to maintaining genomic stability.

**Figure 4:**
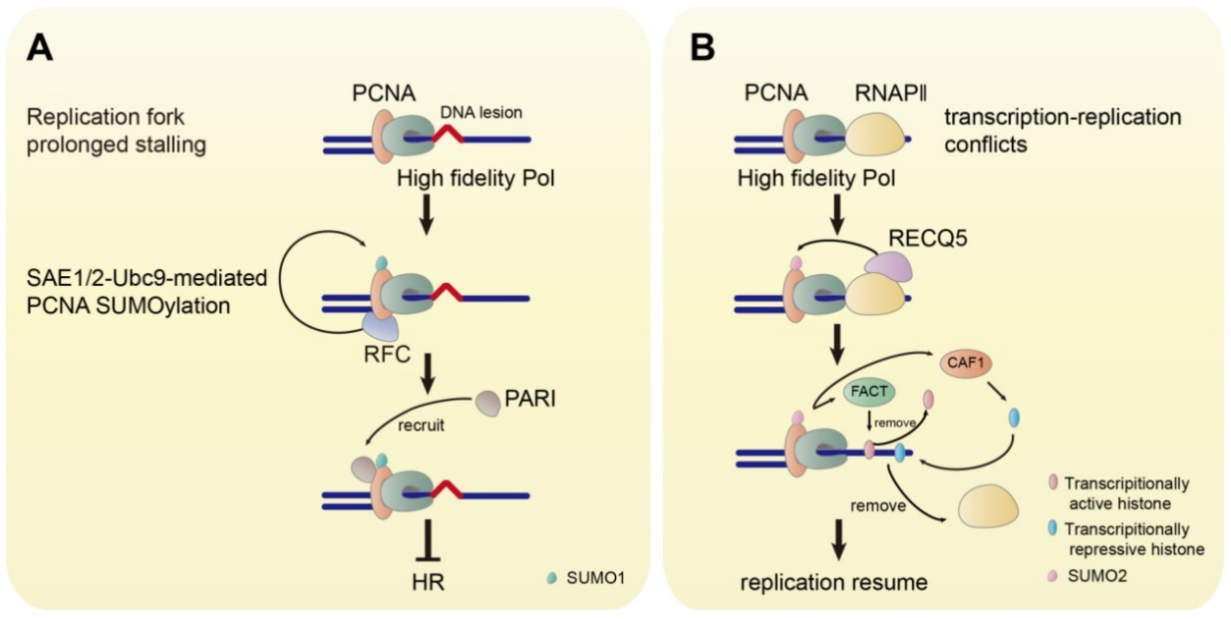
** The Functions of SUMOylated PCNA. (A)** The prolonged stalling of the replication fork induces the SAE1/2-Ubc9-mediated SUMOylation of PCNA by SUMO1. RFC facilitates the SUMOylation of PCNA. The SUMOylated PCNA recruits PARI to inhibit HR. **(B)** The TRC promotes the RECQ5-induced SUMOylation of PCNA by SUMO2. SUMO2-PCNA removes RNAPⅡ by FACT-mediated removal of transcriptionally repressive histone and CAF1-mediated deposition of transcriptionally active histone.

The replication factor C (RFC) is a conserved chaperone-like complex, which loads PCNA onto chromatin. PCNA SUMOylation by SUMO1 is facilitated by RFC. The association of PCNA to RFC is the prerequisite for SUMOylation, while DNA binding is not necessary 84. The human SUMO1-PCNA has also been found to suppress inappropriate HR by recruiting the helicase PARI (Figure 4A) 83. Furthermore, it has been demonstrated that the expression of SUMO1-PCNA fusion protein in human cells inhibits HR in response to DSB and reduces MMS-induced γH2A foci and fragmented double-stranded DNA. The mutation at K164 for PCNA SUMOylation in Rad18^-/-^ cells eliminates the ubiquitination at this site and increases the MMS-induced γH2A foci 84. However, rather than generating DSB directly, MMS-induced DSB derives from replication fork stalling 89. Thus, these observations indicate that the SUMOylation of PCNA by SUMO1 may be critical in preventing the replication fork collapse to DSB to maintain genome stability.

DSB can also occur in unperturbed growing cells induced by transcription-replication conflicts (TRCs). The TRCs cause replication fork stalling leading to replication fork collapse, which results in DSB formation and genomic instability 90, 91. The E3 ligase TRIM28 catalyzes the SUMO2 conjugation to PCNA, which is triggered by transcription to resolve the TRCs 92. The SUMOylation of PCNA by SUMO2 is induced by the RNA polymerase Ⅱ (RNAPII)-bound helicase RECQ5, which suppresses transcription-related DSB and functions as a tumor suppressor 93-97. SUMO2-PCNA temporarily dissociates RNAPⅡ in the collision path by enriching the histone chaperone FACT to remove parental histones ahead of the replication fork and CAF1 to deposit repressive histone marks at the replication site, that ensures the progression of the replication fork avoiding DSB risks (Figure 4B) 97.

The SUMOylation can also serve as a signal for ubiquitination. The SUMO-targeting ubiquitin ligases (STUbLs) harboring SUMO-interaction motifs (SIMs) mediate the ubiquitination of SUMO moieties or SUMOylated proteins 98, 99. The mutant of the K164/K127 of PCNA or SIM of RAD18 reduces PCNA ubiquitination, suggesting that the SUMOylation of PCNA can direct RAD18 to target PCNA 100. This observation implies the crosstalk between SUMOylation and ubiquitination of PCNA.

## Phosphorylation

Phosphorylation, discovered in the mid-1950s, is a principal way to regulate most cellular functions, including protein stability, subcellular localization, and protein-protein interaction. It reversibly confers phosphate group to substrate proteins at a serine, threonine, or tyrosine residue 101.

The chromatin binding is essential for PCNA to exert DNA replication and DDT functions. PCNA is phosphorylated when it binds to DNA synthesis sites 102, 103. In 1995, Zophonias O. Jonsson *et al.* found that the Y211 site of PCNA is critical for chromatin loading, as the Y211A mutation of PCNA completely abolishes its trimer formation, which then cannot be loaded onto DNA by RFC 104. Subsequently, it was found that the Y211 site of PCNA can be phosphorylated by the epidermal growth factor (EGF) receptor (EGFR) to stabilize the chromatin-bound PCNA. The increased PCNA Y211 phosphorylation is frequently correlated to prostate cancer and breast cancer 105, 106. Blocking PCNA Y211 phosphorylation by the Y211F PCNA peptide, the proliferation of prostate cancer and TKI-resistant triple-negative breast cancer (TNBC) cells is significantly restrained 106, 107. The treatment of cells with EGFR inhibitors such as AG1478 or lapatinib suppresses Y211 phosphorylation of PCNA and downregulates chromatin-bound PCNA 108.

In the absence of EGFR, the phosphorylation of PCNA at the Y211 site is also catalyzed by the non-receptor tyrosine kinase c-Abl when the upstream Ron receptor is activated by its ligand hepatocyte growth factor-like protein (HGFL). Imatinib, a pharmacological inhibitor of c-Abl, suppresses the HGFL-induced proliferation of breast cancer cells 88. Thus, the Y211 site of PCNA may be an effective target to develop cancer therapy.

PCNA can also be phosphorylated at Y60, Y133, and Y250 sites by the nuclear IGF-1R (nIGF-1R). The phosphorylation of PCNA leads to the recruitment of E2 conjugating enzyme (UBC13) and E3 ligases (RAD18, SHPRH, and HLTF) for PCNA mono- and poly-ubiquitination. In IGF-1R negative cells, DNA damage induces G1 arrest and S phase replication fork stalling, while the nIGF-1R activation can rescue the stalled replication fork 109. IGF-1R and PCNA are colocalized in many cancer types. The colocalization increases in the tumor tissues compared to the adjacent normal tissues. This colocalization is frequently lost in carcinomas with poor chemotherapy response and a majority of metastatic lesions. However, the stronger colocalization of IGF-1R and PCNA is correlated to the higher overall survival in cancer patients 110.

These findings suggest that the phosphorylation of PCNA is crucial for its loading onto chromatin, which serves as a prerequisite for the succeeding functions of PCNA. Moreover, the phosphorylation may also have crosstalk with the ubiquitination of PCNA.

## Acetylation

The acetylation was first identified on histones in 1964, followed by the discovery of acetylation of non-histone proteins such as high-mobility group (HMG) proteins, tubulin, and p53 111-115. Lysine acetylation is dynamically controlled by two groups of enzymes. The enzymes are lysine acetyltransferases (HATs) and lysine deacetylases (HDACs) 116. The non-histone protein acetylation is implicated in diverse functions, including gene transcription, cell cycle regulation, DNA damage repair, protein stability, protein folding, protein aggregation, cytoskeleton organization, RNA processing, RNA stability, and autophagy 117.

Hoyun Lee and colleagues found that mammalian PCNA is not regulated by phosphorylation but by acetylation. They found that PCNA may be acetylated and deacetylated by p300 and HDAC1, respectively 118. The acetylated PCNA shows a high affinity to Polβ and Polδ, which is important for DNA replication. The acetylation of PCNA at K14 is induced after UV damage and promotes PCNA for degradation to inhibit DNA replication 119. Ennio Prosperi and colleagues further clarified that CBP and p300 are required for the acetylation of PCNA. CBP- and p300-mediated acetylation of PCNA at K13, K14, K77, and K80 promotes the removal of chromatin-bound PCNA and its degradation after DNA synthesis 120. Thus, the acetylation of PCNA avoids the excessive retention of PCNA on chromatin, preventing genomic instability.

In response to MMS-induced DNA damage, the sliding surface of PCNA is acetylated at K20 by Eco1, which stimulates HR and suppresses the DNA damage sensitivity of the DDT mutant yeast cells 121. Depleting HAT3 in *Leishmania donovani* caused the loss of PCNA ubiquitination in response to UV 122. This observation indicates that the HAT3-mediated PCNA acetylation may be a prerequisite for PCNA ubiquitination. This study links the acetylation to the ubiquitination of PCNA in eukaryotes.

## Other PTMs

### Methylation

The understanding of protein methylation is expanding these years since the first report of flagellar protein methylation in 1959. The methyl group is transferred by methyltransferase from *S*-adenosylmethionine (SAM) to lysine, arginine, aspartic acid, or glutamic acid. The methylation is reversible as the methyl group can be removed from substrates by demethylases.

PCNA was found to be methylated at K248 by histone H4K20 methyltransferase SETD8, and this methylation allows the stabilization of PCNA expression 123, 124. SETD8-mediated PCNA methylation increased the interaction between PCNA and flap endonuclease FEN1. This effect is essential for DNA replication and repair. The methylation-inactive mutation of PCNA causes impaired DNA replication and induced DNA damage 123. Depleting SETD8 results in decreased cell proliferation and increased DNA damage during the S phase 125. SETD8 is overexpressed in various types of cancers. The expression of SETD8 and PCNA are correlated in cancer samples, implying the crucial role of SETD8-mediated methylation of PCNA in tumor development 123. Various SETD8 inhibitors have been discovered, such as Nahuoic acid A, UNC0379, SPS8I1-3, MS453 126-129. Among these inhibitors, UNC0379 shows an anti-growth effect on neuroblastoma and ovarian cancer 130, 131. The functional effect of other inhibitors on tumor development and whether they target the regulatory sites of PCNA by SETD8 requires further identification.

The methyltransferase enhancer of zeste homolog 2 (EZH2) is responsible for the tri-methylation of histone H3 at K27 132-134. EZH2 is positively related to cancer cell proliferation and localizes at the replication fork in response to DNA damage 135-138. EZH2 directly binds to PCNA via PIP-box and methylates PCNA at K110. The EZH2-mediated methylation of PCNA stabilizes the PCNA trimer to enhance its interaction with Polδ and DNA replication 139. Inhibitors targeting EZH2 such as 3-Deazaneplanocin A (DZNep), GSK126, EI1, and UNC1999 exhibit a proliferation suppression effect on various tumor cells or cancer models, including Acute Myeloblastic Leukemia (AML), lymphoma, gastric cancer, breast cancer, and MLL-rearranged leukemia 140-145. Additionally, the inhibitor CPI-1205 being in clinical trials has shown a potent anti-tumor effect 146.

### NEDDylation

The neuronal precursor cell-expressed developmentally down-regulated protein 8 (NEDD8) is a small ubiquitin-like molecule. NEDD8 is reversibly conjugated to a lysine residue of target proteins 147. Similar to the ubiquitin system, NEDDylation is also a process triggered by the NEDD8-activating enzyme (E1), NEDD8-conjugating enzyme (E2), and NEDD8 ligase (E3). NEDDylation participates in DNA damage response, such as the decision between non-homologous end joining (NHEJ) and HR 148-150. PCNA can be NEDDylated by RAD18. The deNEDDylation of PCNA is catalyzed by the deNEDDylase NEDP1. In response to H_2_O_2_-induced oxidative stress, NEDP1 dissociates with PCNA causing elevated RAD18-dependent NEDDylation of PCNA. The NEDDylation antagonizes the ubiquitination of PCNA. In addition, the NEDDylation of PCNA disrupts the interaction between PCNA and Polη. The impairment of PCNA NEDDylation reduces the cell sensitivity to oxidative stress and vice versa 151. This crosstalk between NEDDylation and ubiquitination of PCNA may be a mechanism to confine the error-prone TLS to maintain genomic stability.

### ISGylation

ISGylation resembles the ubiquitination process. The interferon-stimulated gene 15 (ISG15) ubiquitin-like modifier is conjugated sequentially by an E1 (ubiquitin-activating enzyme E1-like protein, Ube1L), an E2 (ubiquitin-carrier protein H8, UbcH8), and an E3 (HECT domain and RCC1-like domain-containing protein 5, HERC5, or estrogen-responsive finger protein, EFP) 152-156. The primary function of ISGylation is to impede the infection of intracellular pathogens. ISGylated proteins are also involved in physiological processes such as TLS, autophagy regulation, protein synthesis, cytoskeletal dynamics, and secretion. Moreover, the dysregulation of ISGylation is closely related to cancer proliferation, migration, and sensitivity to chemotherapy 157. Upon UV irradiation, the ISGylation of PCNA is induced by EFP, which binds to mono-ubiquitinated PCNA. The ISGylated PCNA recruits USP10 to deubiquitinate PCNA, which results in Polη disassociation from PCNA. This dissociation leads to the termination of TLS. Ultimately, PCNA is deISGylated by UBP43 for reloading of replicative DNA polymerases to resume normal replication. By ISGylation-defective mutation in PCNA or silencing ISG15/EFP, the compromise of PCNA ISGylation causes persistent retention of PCNA and Polη to nuclear foci. The prolonged recruitment of PCNA and Polη on chromatin increases mutation frequency 77. This investigation indicates that ISGylation of PCNA is essential for controlling the appropriate TLS and preventing mutagenesis.

## Conclusions and Perspectives

PCNA is the master of DNA replication and DDT during the S phase. The faithful replication and timely lesion bypass if the replication fork encounters lesions are indispensable events for cell survival and proliferation. PCNA trimer serves as a coordinator recruiting DNA polymerases and slides on the DNA to facilitate DNA synthesis. When replication fork stalls induced by endogenous or exogenous sources of damage, PCNA is ubiquitinated and transfers to bind DDT DNA polymerases or mediate template switching. This process ensures the replication processivity. The ubiquitination of PCNA is removed by DUBs to restrict unwanted mutagenesis 69, 71, 75. Thus, the appropriate regulation of PCNA in the control of DNA replication and DDT is critical for normal cellular function and preventing mutagenesis.

PTMs dynamically regulate the functions of target proteins to fine-tune biological processes in response to environmental changes. In this review, we discuss the regulation and functions of PCNA PTMs. PCNA can be modified by ubiquitination, SUMOylation, phosphorylation, acetylation, methylation, NEDDylation, and ISGylation (Table 1), and the modification sites are presented in Figure 1. Chromatin loading is a requisite for PCNA to exert replication and repair functions. PCNA phosphorylation catalyzed by EGFR or c-Abl is critical for PCNA loading onto chromatin 88, 105. The affinity to Polδ, responsible for DNA replication, is regulated by PCNA acetylation 118. The acetylation of PCNA also promotes its degradation to suppress DNA synthesis in response to DNA damage 119, which may provide time for DNA damage response and repair.

When the replication fork stalls, the ubiquitination allows PCNA to recruit TLS polymerases or promote TS to tolerate the DNA lesions. The DNA binding of PCNA and the histones are essential for the ubiquitination of PCNA 41, 47. Several DNA damage response factors (WRN, PTIP, and RNF8) and kinases (IGF-1R and Akt) related to cell growth signal function in regulating PCNA ubiquitination 51, 59-61, 64, 68.

The SUMOylation of PCNA with SUMO1 is regulated by RFC 84. The SUMO1-PCNA negatively regulates HR to suppress genome rearrangements and DSB 83, 86. The SUMO2 is conjugated to PCNA by TRIM28 E3 ligase and induced by RECQ5 92, 97. The SUMO2-PCNA ensures replication progression by resolving the collision between transcription and replication machinery 97.

SETD8 and EZH2, which are positively correlated to tumor development, can catalyze PCNA methylation. The methylation causes PCNA stabilization and increases its interaction with FEN1 and Polδ for DNA replication and DDT 123, 139. The NEDDylation of PCNA is dynamically regulated by the E3 ligase RAD18 and the deNEDDylase NEDP1. The NEDDylation of PCNA impairs the association between PCNA and Polη 151, which may be essential to control TLS.

The modification crosstalk also takes place between the SUMOylation and the ubiquitination of PCNA. The SUMOylation serves as a signal for PCNA ubiquitination, as it activates RAD18 to target SUMO-PCNA 100. The nuclear IGF-1R-mediated phosphorylation of PCNA promotes its ubiquitination by recruiting UBC13, RAD18, SHPRH, and HLTF. The colocalization of PCNA and IGF-1R is deficient in cancer tissues from patients with poor chemotherapy response, suggesting that the crosstalk between phosphorylation and ubiquitination of PCNA may predict the high overall survival of cancer patients 110. PCNA ubiquitination is deficient in acetyltransferase HAT3-null *Leishmania donovani* 122. This result indicates that acetylation may promote the ubiquitination of PCNA. In contrast, NEDDylation suppresses the ubiquitination of PCNA in response to oxidative stress 151, which suggests a negative regulation of the NEDDylation for ubiquitination of PCNA. In addition, ISGylation antagonizes the ubiquitination of PCNA directly. The ISGylated PCNA recruits USP10 to reduce the ubiquitination of PCNA and leads to the termination of TLS subsequently 77.

The PTMs of PCNA and the related regulators described above have several implications in carcinogenesis. These modifications can be aberrantly utilized by cancer cells to exert unlimited growth and resistance to chemotherapy (Table 2). Consequently, elucidating the regulation and mechanism of PCNA PTMs will provide insights to develop cancer therapies. As summarized in Table 3, the Y211F PCNA peptide targeting the phosphorylation site Y211 of PCNA inhibits the proliferation of prostate cancer and TNBC cells effectively 106, 107. A variety of small molecular inhibitors against the enzymes or regulators of PCNA PTMs, including SMI#9 (RAD6, ubiquitination), AG1478 (phosphorylation, EGFR), and Lapatinib (phosphorylation, EGFR), have been identified that they suppress cell proliferation or enhance chemosensitivity via inhibition of these PCNA PTMs 35-38. Although WRN, c-Abl, SETD8, and EZH2 show regulation of PCNA PTMs (Table 1) and potent anti-growth effects on various tumors (Table 2), the anti-tumor mechanisms directly correlated to the regulation of PCNA PTMs need further investigation. Moreover, novel inhibitors or specific blocking peptides suppressing tumor development via inhibition of PCNA PTMs require identification.

According to these investigations, the regulation outline of PCNA PTMs gradually becomes clear. The modulations and the crosstalk in these PTMs of PCNA orchestrate its functions in DNA replication and DDT. However, the dysregulation of PCNA PTMs will lead to mutagenesis and ultimately result in carcinogenesis. Thus, clarifying the mechanisms of PCNA PTMs is critical for understanding tumor development, which may provide effective targets for cancer therapy.

## Figures and Tables

**Table 1:** PTMs and the regulators of PCNA

PTM	enzyme	site	regulator	Function
Ubiquitination	Rad6-Rad18; Rad5-Ubc13-Mms2	K164	free RPA	inhibition of free PCNA mono-ubiquitination (ref. 41)
			DNA-bound RPA	promotion of chromatin-bound PCNA mono-ubiquitination (ref. 41, 43)
			H3/H4	promotion of PCNA ubiquitination (ref. 47)
			WRN	inhibition of PCNA ubiquitination (ref. 51)
			PTIP	promotion of PCNA ubiquitination (ref. 59)
			IGF-1/IGF-1R	inhibition of PCNA ubiquitination (ref. 64)
			Akt	promotion of PCNA ubiquitination (ref. 68); promotion of chromatin recruitment of Polη (ref. 68)
	RNF8-UbcH5c/Ubc13/Uev1a	K164		promotion of PCNA ubiquitination (ref. 60)
SUMOylation	SAE1/SAE2, Ubc9, Pias1-4	K164	RFC	promotion of SUMO1-PCNA (ref. 84)
	TRIM28	K164	RECQ5	promotion of SUMO2-PCNA (ref.^92^, ^97^)
Phosphorylation	EGFR	Y211		stabilization of the chromatin-bound PCNA (ref. 105, 88)
	c-Abl			
	nIGF-1R	Y60, Y133, Y250		rescue stalled replication fork (ref. 109); promotion of PCNA ubiquitination (ref. 109)
Acetylation	CBP, p300	K13, 14, 77, 80		chromatin-bound PCNA removal (ref. 120); PCNA degradation (ref. 120)
	Eco1	K20		HR stimulation (ref. 121)
	HAT3			promotion of PCNA ubiquitination (ref. 122)
Methylation	SETD8	K248		stabilization of PCNA expression (ref. 123); promotion of interaction between PCNA and FEN1 (ref. 123)
	EZH2	K110		stabilization of PCNA trimer (ref. 139); promotion of interaction between PCNA and Polδ (ref. 139)
NEDDylation	RAD18			disruption of interaction between PCNA and Polη (ref. 151); inhibition of PCNA ubiquitination (ref. 151)
ISGylation	EFP			inhibition of PCNA ubiquitination (ref. 77); Polη dissociation from PCNA (ref. 77)

**Table 2:** The implications of PCNA PTMs in carcinogenesis

PTM	Site in PCNA	Implication in Carcinogenesis	Reference
Ubiquitination	K164	RAD6, RAD18→development and therapy resistance of tumors.	26-36
		H3/H4→MMS-induced mutagenesis.	47
		WRN mutation→predisposition to cancer	49
		RNF8→cell cycle arrest and apoptosis of medulloblastoma cells	61
		IGF signal→cell proliferation, survival, apoptosis, differentiation, metabolism, migration	63
SUMOylation	K164, K127	SUMO1-PCNA→prevention of the replication fork collapse to DSB	83, 84, 89
		SUMO2-PCNA→avoidance of the TRCs-induced DSB	90-92, 97
Phosphorylation	Y211	Increased phosphorylation→prostate and breast cancer	105, 106
	Y60, Y133, Y250	IGF-1R and PCNA colocalization→many cancer types, lost in tumors with poor chemotherapy response, high overall survival	109, 110
Acetylation	K13, K14, K77, K80	Removal of chromatin-bound PCNA→avoidance of excessive retention of PCNA and genomic instability	120
Methylation	K248	SETD8→cell proliferation, high expression in many types of cancers	125, 123
	K110	EZH2→cancer cell proliferation	135-138
NEDDylation		Cell sensitivity to oxidative stress, maintain genomic stability	151
ISGylation		Control of appropriate TLS and prevention of mutagenesis	77, 157

**Table 3:** Inhibitors or blocking peptides relating to PTMs and regulators for cancer therapy

Inhibitor	Affected PTM	Target	Cancer Type/Cancer Cell Type	Reference
SMI#9	Ubiquitination	RAD6	breast cancer, ovarian cancer	35-38
NSC19630	Ubiquitination	WRN	HeLa cells	52
Y211F PCNA peptide	Phosphorylation	Y211 site in PCNA	prostate cancer, breast cancer	106, 107
AG1478, Lapatinib	Phosphorylation	EGFR	MDA-MB-468 cells	108
Imatinib	Phosphorylation	c-Abl	breast cancer	88
UNC0379	Methylation	SETD8	neuroblastoma, ovarian cancer	130, 131
DZNep, GSK126, EI1, UNC1999, CPI-1205	Methylation	EZH2	AML, lymphoma, gastric cancer, breast cancer, MLL-rearranged leukemia	140-146

## Abbreviations

PTMs: post-translational modifications
PCNA: proliferating cell nuclear antigen
TLS: translesion synthesis
TS: template switching
ROS: reactive oxygen species
UV: ultraviolet
IR: ionizing radiation
DSB: DNA double-strand break
DDT: DNA damage tolerance
PIP: PCNA-interacting protein box
K: lysine
DUBs: deubiquitination enzymes
HLTF: helicase like transcription factor
SHPRH: SNF2 histone linker PHD RING helicase
RPA: replication protein A
MMS: methyl methanesulfonate
WS: Werner Syndrome
IGF: insulin-like growth factor
IGF-1 receptor: IGF-1R
HR: homologous recombination
SUMO: small ubiquitin-related modifier
SUPs: SUMO-specific proteases
RFC: replication factor C
STUbLs: SUMO-targeting ubiquitin ligases
SIMs: SUMO-interaction motifs
TRCs: transcription-replication conflicts
RNAPⅡ: RNA polymerase Ⅱ
EGF: epidermal growth factor
EGFR: EGF receptor
TNBC: triple-negative breast cancer
HGFL: hepatocyte growth factor-like protein
nIGF-1R: nuclear IGF-1R
HMG: high-mobility group
HATs: lysine acetyltransferases
HDACs: lysine deacetylases
SAM: *S*-adenosylmethionine
EZH2: zeste homolog 2
DZNep: 3-Deazaneplanocin A
AML: Myeloblastic Leukemia
NEDD8: neuronal precursor cell-expressed developmentally down-regulated protein 8
NHEJ: non-homologous end joining
ISG15: interferon stimulated gene 15
